# Evidence on nutritional therapy practice guidelines and implementation in adult critically ill patients: A systematic scoping review

**DOI:** 10.4102/curationis.v42i1.1973

**Published:** 2019-12-13

**Authors:** Nomaxabiso M. Mooi, Busisiwe P. Ncama

**Affiliations:** 1 School of Nursing and Public Health, College of Health Sciences, University of KwaZulu-Natal, Durban, South Africa

**Keywords:** critically ill adults, nutritional therapy, practice guidelines, implementation, enteral nutritional therapy protocol, parenteral nutritional therapy protocol, algorithm, scoping review

## Abstract

**Background:**

The rapid increase in disease-related malnutrition makes it almost impossible for healthcare practitioners and policymakers to keep up with its negative consequences. Consequently, healthcare organisations and decision-makers have called for accelerated and double-duty actions to manage the double burden of malnutrition. Guidelines standardise nutritional practices, improve nutritional status and reduce hospitalisation duration and save costs.

**Objectives:**

A systematic scoping review of the nutritional therapy practice guidelines and implementation in critically ill adults was undertaken to identify the breadth of literature on the topic, summarise findings and identify gaps.

**Methods:**

A comprehensive search strategy was designed and implemented to identify eligible studies from eight databases, websites of organisations, government departments and academic platforms. Reference lists of included studies were also searched for relevant studies. We assessed the quality of included studies, completed a descriptive numerical summary and analysed them.

**Results:**

In total, 1555 titles and 101 abstracts were screened, 65 underwent full text review and 19 were retained for data extraction. Studies scored average to high on quality assessment, and a summary of characteristics of included studies is presented. Nutritional therapy practice guidelines are considered a proactive strategy for enhanced, uniform and individualised nutritional practices and factors that influence implementation were identified.

**Conclusions:**

A gap exists between research recommendations and actual practice despite the growing interest in implementation of nutritional therapy guidelines in critical care. There is a need for more research to evaluate the practicality of available guidelines.

## Background

The quadruple burden of communicable and non-communicable diseases, perinatal and maternal conditions, and injury-related disorders has resulted in a double burden of malnutrition, which is defined as the combination of over- and undernutrition (Mayosi & Benatar 2014:1345; WHO 2017:1). People live longer because of modern treatment of these diseases, and many are either chronically underweight or overweight and therefore are vulnerable to acute illness (National Collaborating Centre for Acute Care 2006:2; WHO 2017:1). This poses a real and growing global health challenge that warrants urgent nutrition actions, and puts pressure on acute care services, which include critical care (Hirshon et al. 2013:386; WHO 2016, 2017:6).

Malnutrition is very common in acutely ill patients, occurring in 30% – 50% of hospitalised patients and the number may be higher in critically ill patients, and is associated with increased complication risk, high healthcare costs and increased long-term mortality (Jones et al. 2008:301; Wischmeyer 2013:2). This is attributed to critically ill patients having decreased volitional nutrition intake and therefore being completely dependent on their care providers for their nutritional needs. The phrase ‘critically ill’ refers to patients ≥18 years of age, with a high severity of disease score, with one or more organ dysfunctions and needing a single or multiple invasive therapeutic intervention(s) (Boniatti et al. 2011). These patients always receive poor nutrition for a prolonged period as nutritional therapy is often only an afterthought on care rounds in most severely ill patients (Wischmeyer 2013:2).

Optimal nutritional therapy has become an important component in the management of critical illness. It is relatively inexpensive compared to other commonly used treatments and is a determinant of quality in patient care (Bousie et al. 2016:e47; Padilla Fuentes et al. 2016:1219; Ridley, Gantner & Pellegrino 2015:565; Warren, McCarthy & Roberts 2016:334). However, despite its important benefits, nutritional practices in critically ill patients remain widely varied, leading to inappropriate and suboptimal nutrition delivery with harmful consequences (Ridley et al. 2015:365). As a result, critically ill patients receive only about 50% of prescribed nutrition in the first 2 weeks following intensive care unit (ICU) admission (Peev et al. 2015:21). Many studies recommend the development and implementation of nutritional therapy guidelines to assist and improve nutritional practices (Bousie et al. 2016:e47; Keller et al. 2015:1; Kim et al. 2017:27). In this study, nutritional therapy is referred to as nutritional support, which will be either enteral nutrition (EN) or parenteral nutrition (PN). Various operational versions of guidelines adapted to local requirements and easy application in clinical practice such as protocols, algorithms and clinical practice guidelines (CPGs) are used to enhance international guidelines implementation (Friesecke et al. 2014:204; Kiss et al. 2012; Mesejo et al. 2011:2).

Guidelines are developed to assist practitioner and patient decisions about appropriate healthcare for specific clinical circumstances, are designed to minimise variation, improve costs and improve outcomes (Dhaliwal et al. 2014:29). They offer basic recommendations supported by review and analysis of the current literature, other national and international guidelines and a blend of expert opinion and clinical practicality (McClave et al. 2016:159; Taylor et al. 2016:391). Additionally, the important aspect of any guidelines is their transparency, that is, the methodical review which links the recommendation to the supporting evidence is clearly stated (Martindale, McCarthy & McClave 2011:463). They should be interpreted and used through the scope of individual institutional practices, and should be based on individual preferences, patient mix, and local expertise. Thus, an effective guideline is clinically practical (Martindale et al. 2011:463). The aim of this review was to map the literature on the availability and implementation of nutritional therapy guidelines in critically ill adults. It is expected that the results of this study may help identify gaps and indications for future researches on the topic.

## Methodology

We followed Arksey and O’Malley’s scoping review methodological framework, adapted by Levac, Colquhoun and O’Brien (Arksey & O’Malley 2005; Levac et al. 2010), with the following stages.

Stage 1: Identifying the research question

Stage 2: Identifying relevant studies

Stage 3: Study selection

Stage 4: Charting the data

Stage 5: Collating, summarising and reporting the results

Stage 6: Consultation (optional).

### Identifying the research question

The main objective of this scoping review was to map the literature on the extent and implementation of nutritional therapy guidelines among critically ill adults, describe key findings and identify emerging themes. Therefore, the broad question the review asked was, ‘what evidence exists on nutrition therapy guidelines and implementation in adult critically ill patients?’

#### Determining the eligibility of the review question

To determine the eligibility of the research question, the Population, Concept, Context (PCC) framework was followed as shown in Table 1.

**TABLE 1:** Eligibility criteria for the review question. A PCC framework for determination of eligibility of review question.

Variable	Description
Population	This study includes researches reporting on adult critically ill patients, critically ill individuals of ≤ 18 years of age, intensive care unit (ICU) patients and critically ill persons. Critically ill neonates, children and individuals below 18 years of age were excluded.
Concept	This review included studies reporting on nutritional therapy guidelines, nutritional policy for enteral and parenteral nutrition, nutritional support guidelines, nutrition clinical practice guidelines, enteral nutrition practice guidelines, parenteral nutrition practice guidelines, artificial feeding guidelines, nutritional practice recommendations, standardised nutritional practices, nutritional therapy protocols, guides, procedures and algorithms. Studies reporting on oral or normal diet were not included.
Context	The scoping review considered studies that discussed the implementation of, compliance with or adherence to enteral or parenteral nutritional therapy guidelines in the management of critically ill patients. Studies from any geographic setting will be eligible for inclusion. Evidence not focusing on or including nutrition therapy guidelines implementation, or effectiveness were excluded.

PCC, Population, concept, context.

### Identifying relevant studies and grey literature

The purpose of the study guided the scoping review to identify published and unpublished studies and reviews suitable for answering the central research question. A comprehensive search was conducted in PubMed, Google Scholar, EBSCO databases, namely Cumulative Index for Nursing and Allied Health Literature (CINAHL), Medline Psych INFO, Psych ARTICLES, Health Source-Consumer Edition and Health Source: Nursing/Academic Edition. Relevant organisations such as the World Health Organization international and academic sources from Open Access for Theses and Dissertations provided grey literature. Keywords and MeSH terms were used to search for studies, guided by the predetermined PCC format and research question. These were separated or combined into phrases by Boolean terms such as ‘OR’ and ‘AND’ and included ICU OR critically ill adults, nutrition therapy OR nutritional support AND guidelines. The initial search strategy that was piloted on the PubMed database, which was adapted and used for other databases (see Table 2), includes the following:

(‘ICU’) OR (‘critical illness’ OR (‘critical’[All Fields AND ‘illness’[All Fields]) OR ‘critical illness’[All Fields] OR (‘critically’[All Fields] AND ‘ill’[All Fields]) OR ‘critically ill’[All Fields]) AND (‘adult’[MeSH Terms] OR ‘adult’[All Fields] OR ‘adults’[All Fields])) AND ((‘nutritional support’[MeSH Terms] OR (‘nutritional’[All Fields] AND ‘support’[All Fields]) OR ‘nutritional support’[All Fields] OR (‘nutrition’[All Fields] AND ‘therapy’[All Fields]) OR ‘nutrition therapy’[All Fields] OR ‘nutrition therapy’[MeSH Terms] OR (‘nutrition’[All Fields] AND ‘therapy’[All Fields]) AND (‘guideline’[Publication Type] OR ‘guidelines as topic’[MeSH Terms] OR ‘guidelines’[All Fields]).

**TABLE 2:** Search terms per database.

Search date	Database	Keywords/link	Articles retrieved	Eligible titles
February 19, 2018	PubMed	Critically ill adults AND nutritional therapy guidelines: ((‘ICU’) OR (‘critical illness’ OR (‘critical’[All Fields] AND ‘illness’[All Fields]) OR ‘critical illness’[All Fields] OR (‘critically’[All Fields] AND ‘ill’[All Fields]) OR ‘critically ill’[All Fields]) AND (‘adult’[MeSH Terms] OR ‘adult’[All Fields] OR ‘adults’[All Fields])) AND ((‘nutritional support’[MeSH Terms] OR (‘nutritional’[All Fields] AND ‘support’[All Fields]) OR ‘nutritional support’[All Fields] OR (‘nutrition’[All Fields] AND ‘therapy’[All Fields]) OR ‘nutrition therapy’[All Fields] OR ‘nutrition therapy’[MeSH Terms] OR (‘nutrition’[All Fields] AND ‘therapy’[All Fields])) AND (‘guideline’[Publication Type] OR ‘guidelines as topic’[MeSH Terms] OR ‘guidelines’[All Fields])	224	39
February 19, 2018	Google Scholar	ICU OR critically ill OR critical illness AND adults, nutrition OR feeding OR food, nutritional support OR nutritional therapy AND guidelines OR protocols OR algorithms OR evidence-based recommendations OR standard practice OR standardised practices	764	25
February 19, 2018	EBSCO host databases: MEDLINE with Full Text CINAHL Health Source: Nursing/Academic Edition PsychINFO Health Source-Consumer Edition PsychARTICLES	(ICU patients OR critically ill patients OR critical illness AND adults OR adult patients) AND (nutrition OR feeding OR food, enteral nutrition, parenteral nutrition, nutritional support OR nutritional therapy AND guidelines OR protocols OR algorithms OR evidence-based recommendations OR standard practice OR standardised practices) (nutrition OR feeding OR food, enteral nutrition, parenteral nutrition, nutritional support OR nutritional therapy guidelines OR protocols OR algorithms OR evidence-based recommendations OR standard practice OR standardised practices)	541	66
February 21, 2018	Grey Literature: WHO International sources; Governmental websites; Open Access for Theses and Dissertations	Intensive care unit OR critically ill adults nutrition therapy AND guidelines	26	24
**Total**			**1555**	**154**

ICU, intensive care unit.

### Selection criteria

#### Inclusion criteria

Studies were included if they were published between January 2002 and February 2018 and discussed critically ill or ICU adults. The start date of 2002 was chosen because many nutritional therapy practice guidelines were published around that period. Studies reporting on nutritional therapy, nutritional support, enteral and parenteral nutrition were included. Guidelines included any document designed to guide nutritional therapy practices or facilitate implementation of guidelines, namely, protocols, evidence-based recommendations, algorithms, nutrition standard practices and reference documents. All study designs such as qualitative, quantitative and mixed methods studies were deemed eligible.

#### Exclusion criteria

For the purpose of this review, we excluded studies reporting on critically ill adults on normal diet and critically ill patients younger than 18 years of age. Evidences from nutritional therapy guidelines in other groups other than critically ill adults were also excluded. Nutritional practice guidelines not focusing on enteral or parenteral nutritional therapy were not included. Conference abstracts, conference presentations, letters to editors and others, such as commentaries, editorials and studies published outside the preferred date range, were not included.

#### Study selection

**FIGURE 1:**
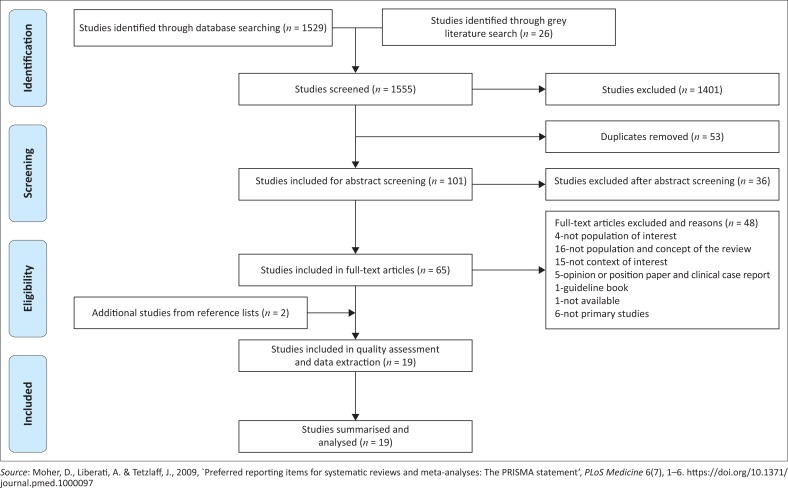
PRISMA flow diagram showing literature search and selection of studies.

The process involved three phases, namely title, abstract and full-text article screening using forms that were developed before the screening commenced. The first phase involved title screening, exporting them onto an EndNote library and removal of duplicates. Abstracts were obtained for included titles, and those articles for which abstracts were not available were included for abstract screening if the title was enough to indicate that nutritional therapy guidelines and implementation were discussed. Two independent reviewers screened abstracts and full text articles. For all articles that we considered relevant after title and abstract screening, we obtained full text articles and included them for data extraction. We resolved our disagreements by discussion, and a third reviewer was invited when necessary. SPSS version 25 was used to calculate Kappa’s statistic to estimate the degree of agreement between reviewers. Reference lists of included studies were also searched to ensure that we considered all possible relevant articles. The results of study selection are shown in shown in Figure 1, adopted and adapted by the researcher (Liberati et al. 2009:4; Moher, Liberati & Tetzlaff 2009:3).

### Data extraction

A pretested data extraction form as described by Noyes and Lewin (2011) was used to extract data from included studies. The form was piloted on the first five studies to ascertain that it was in line with the research question. We extracted data relevant to nutritional therapy practice guidelines implementation by author, year of publication, country of origin, study aim, study setting, relevant results, significant results and authors’ conclusions. Table 3 shows a summary of included studies.

**TABLE 3:** Summary of included studies.

Characteristic	Number (*n* = 19)	%
**Publication year**		
2004–2008	5	26
2009–2012	4	21
2013–February 2018	10	53
**Guideline terminology**		
Clinical practice guideline (CPG)	6	32
Protocol	9	47
Algorithm	4	21
**Setting**		
Tertiary hospital ICU	11	58
University or teaching hospital	9	47
General hospital	1	5
National Department of Health	3	16
Not specified	4	21
**Aim or focus of the study**		
Effects of guideline implementation	13	68
Rate of guideline implementation and other	6	32
**Study design**		
Retrospective	7	37
Prospective	9	47
Not specified	3	16
**Guideline type**		
Enteral Nutritional therapy (EN)	6	32
Parenteral Nutritional therapy (PN)	1	5
EN and PN	12	63

ICU, intensive care unit.

#### Quality assessment

Reviewers met to choose quality appraisal tools for included studies from both published (black) and unpublished (grey) literature. The Mixed Method Appraisal Tool (MMAT) version 2011 was employed to assess methodological quality of included primary studies (Pluye et al. 2011). The tool is designed to assess studies according to study design types or domains, namely all study types, quantitative randomised controlled and quantitative non-randomised and quantitative descriptive (Pace et al. 2012:47). No studies used qualitative, quantitative, descriptive or mixed methods, and no studies were excluded because of low quality. The scoring merits ranged between 25% and 100%. A score of 25% – 50% was rated as low quality, 51% – 75% as average and 76% – 100% as high quality.

A different tool, the authority, accuracy, coverage, objectivity, date and significance (AACODS) checklist was used to evaluate and critically appraise grey literature. The checklist comprises 28 items that are unevenly distributed under six domains. The tool did not come with scores, and scoring metrics were developed by the reviewers. In order to provide a quantitative estimate of the overall quality score, each item was allocated 3.6 points, which gave a total of 100 when multiplied by 28 (number of items). The overall quality score was set at 10 and the score index was 0.1–1; a study with a score of four (40 points) or less was rated as weak, a study scoring between five and seven was rated moderate, while a score of eight and higher was considered strong. The summary of the quality assessment report of grey literature studies is shown in Table 4.

**TABLE 4:** Summary of quality assessment report of included studies from databases (MMAT 2011).

Author and date	Are there clear qualitative and quantitative or mixed methods research questions (or objectives)?	Do the collected data address the research question (objective)?	Quantitative non-randomised controlled trials				Score %
			Are participants recruited in a way that minimises selection bias?	Are measurements appropriate regarding exposure/intervention and outcomes?	Are participants comparable or difference between these groups taken into account?	Are there complete outcome data (80% or above)	
Barr et al. 2004	Yes	Yes	Yes	Yes	Yes	Yes	100
Bousie et al. 2016	Yes	Yes	Yes	Yes	Yes	Yes	100
Cahill et al. 2010a	Yes	Yes	Yes	Yes	Yes	Yes	100
Compton et al. 2014	Yes	Yes	Yes	Yes	Yes	Yes	100
Dervan et al. 2012	Yes	Yes	Yes	Yes	Yes	Yes	100
Dobson and Scott 2007	Yes	Yes	Yes	Yes	No	Yes	75
Heyland et al. 2004	Yes	Yes	Yes	Yes	No	Yes	75
Heyland et al. 2015	Yes	Yes	Yes	Yes	Yes	Yes	100
Kim et al. 2017	Yes	Yes	Yes	Yes	Yes	Yes	100
Kiss et al. 2012	Yes	Yes	Yes	Yes	Yes	Yes	100
Mackenzie et al. 2005	Yes	Yes	Yes	Yes	Yes	Yes	100
Pasinato et al. 2013	Yes	Yes	Yes	Yes	No	Yes	75
Quenot et al. 2010	Yes	Yes	Yes	Yes	Yes	No	75
Sungur et al. 2015	Yes	Yes	Yes	Yes	Yes	Yes	100
Wang et al. 2017	Yes	Yes	Yes	Yes	Yes	Yes	100
Wøien and Bjørk 2006	Yes	Yes	Yes	Yes	Yes	Yes	100

### Collating, summarising and reporting results

The goal of this scoping review is to analyse eligible studies to obtain an overview of the scientific literature on availability and implementation of nutritional therapy guidelines in critically ill adults. With this goal in mind, we summarised and presented the collection of key findings on the subject matter from eligible studies. Responses to these questions were collated, summarised and reported as results of our scoping review. Results of our study include both a descriptive numerical summary of the characteristics of included studies and a thematic analysis, based on the questions we asked during the charting process. Thematic analysis as described by Braun and Clarke (2006) was used to analyse qualitative data from the results sections of included studies. Results were collated, summarised and coded. The code list generated across the data sets was collated, sorted and searched for potential themes. Identified themes were then reviewed in search for final themes, which were then defined and named. Table 5 shows the themes that were generated.

**TABLE 5a:** Summary of quality assessment report of included studies from grey literature (AACODS).

Criteria	Description	IrSPEN Special Report No. 1, 2013	National DoH, EN, 2016	National DoH, PN, 2017
Authority	Is the organisation reputable?	1	1	1
	Is the organisation an authority in the field?	1	1	1
	Does the item have a detailed reference list or bibliography?	1	1	1
		10.8	10.8	10.8
Accuracy	Does the item have a clearly stated aim or brief?	1	1	1
	If so, is this met?	1	1	1
	Does it have a stated methodology?	1	0	0
	If so, is it adhered to?	1	0	0
	Has it been peer-reviewed?	0	0	0
	Has it been edited by a reputable authority?	0	0	0
	Supported by authoritative, documented references or credible sources?	1	1	1
	Is it representative of work in the field?	1	1	1
	If No, is it a valid counterbalance?	0	0	0
	Is any data collection explicit and appropriate for the research?	0	0	0
	If the item is a secondary material (e.g. a policy brief of a technical report), refer to the original.	1	1	1
	Is it an accurate, unbiased interpretation or analysis?	1	0	0
		28.8	18.0	18.0
Coverage	Are any limits clearly stated?	0	1	1
		0	0	0
Objectivity	Opinion, expert or otherwise, is still opinion: is the author’s standpoint clear?	1	1	1
	Does the work seem to be balanced in presentation?	1	1	1
		7.2	7.2	7.2
Date	Does the item have a clearly stated date related to content?	1	1	1
	If no date is given, but can be closely ascertained, is there a valid reason for its absence?	0	0	0
	Check the bibliography: have key contemporary materials been included?	1	1	1
		7.2	7.2	7.2
Significance	Is the item meaningful? (this incorporates feasibility, utility and relevance)	1	1	1
	Does it add context?	1	1	1
	Does it enrich or add something unique to the research?	1	1	1
	Does it strengthen or refute a current position?	1	1	1
	Would the research area be lesser without it?	1	1	1
	Is it integral, representative, typical?	1	1	1
	Does it have impact? (in the sense of influencing the work or behaviour of others)	1	1	1
		25.2	25.2	25.2
**Total scores**		**79.2**	**69.4**	**69.4**

**TABLE 5b:** Summary of quality assessment report of included studies from grey literature (AACODS).

Author/organisation	Country	Quality score	Quality index score
IrSPEN Special Report No. 1, 2013	Ireland	79.2	0.79
National DoH, EN, 2016	South Africa	69.4	0.69
National DoH, PN, 2017	South Africa	69.4	0.69

EN, enteral nutritional therapy; PN, parenteral nutritional therapy.

### Ethical considerations

The study is a review of literature and does not involve human participants.

### Results

Eight databases were searched along with World Health Organization (WHO) International and Open Access for Theses and Dissertations (grey literature) and 1555 articles were identified. Following screening, 19 studies met the inclusion criteria (Figure 1). The degree of agreement between the reviewers was 80.0%, while the Kappa statistics was 0.58, *p* < 0.001, which showed moderate to substantial level of agreement. McNemar’s chi-square statistics suggests that there is no statistical significant difference in the proportion of ‘yes’ or ‘no’ reported by the screeners. Forty-eight studies were excluded at full text article screening stage with reasons. Thirty-five of these studies failed to meet the inclusion criteria; five were opinion papers or editorial letters, six of them were secondary studies, and two were excluded because one was a guideline booklet and one was not available.

## Characteristics of included studies

**FIGURE 2:**
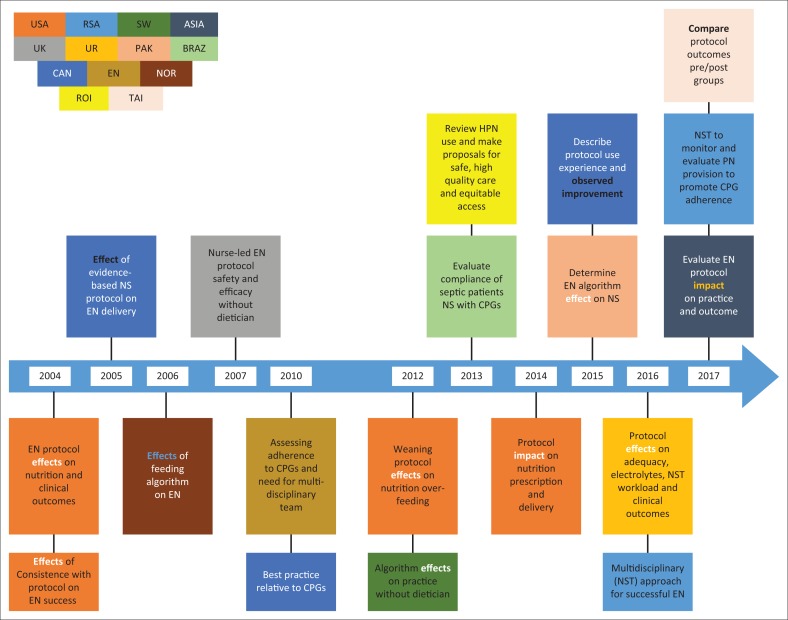
Summarised focus of included studies.

A summary of descriptive characteristics of included studies is provided in Table 6. The studies were published between 2004 and 2017, with the majority appearing in the year preceding the scoping review, 2017 (Figure 2). Studies originated from 13 countries: four are from the USA (Barr et al. 2004; Compton et al. 2014; Dervan et al. 2012; Heyland et al. 2004); three from Canada (Cahill et al. 2014; Heyland et al. 2015; Mackenzie et al. 2005); South Africa (Africa 2016; National Department of Health 2017) and Brazil (Pasinato et al. 2013) published two studies each; and the remaining eight studies are from researchers located in Europe (Bousie et al. 2016), Pakistan (Sungur, Sahin & Tasci 2015), United Kingdom (Dobson & Scott 2007), Taiwan (Wang et al. 2017), Norway (Wøien & Bjørk 2006), Ireland (Rice Niamh 2013), England (Quenot et al. 2010), Switzerland (Kiss et al. 2012) and Asia (Kim et al. 2012). Fourteen primary studies followed the prospective (Barr et al. 2004; Cahill et al. 2010b; Dobson & Scott 2007; Heyland et al. 2004; Pasinato et al. 2013; Quenot et al. 2010; Windle 2009; Wøien & Bjørk 2006) and retrospective research designs (Bousie et al. 2016; Compton et al. 2014; Kim et al. 2017; Kiss et al. 2012; Mackenzie et al. 2005; Wang et al. 2017), eight and six, respectively, two were multicentre quality improvement initiatives (Heyland et al. 2015; Quenot et al. 2010) and one was a quasi-experimental study (Sungur et al. 2015). With regard to studies from grey literature, two of them (National Department of Health 2016; National Department of Health 2017) were guideline documents based on literature review and expert opinion from South Africa, and one was a secondary analysis of a prospective observational cohort study data from Ireland (Rice Niamh 2013). Of the 19 studies, 10 studies discussed guideline implementation on mechanically ventilated critically ill adults (Bousie et al. 2016; Cahill et al. 2010a; Compton et al. 2014; Dervan et al. 2012; Heyland et al. 2004; 2015; Quenot et al. 2010; Rice Niamh 2013; Sungur et al. 2015; Wang et al. 2017). Eleven of the included primary studies were conducted in tertiary institutions of which nine were university or teaching hospitals (Barr et al. 2004; Bousie et al. 2016; Cahill et al. 2010a; Compton et al. 2014; Dervan et al. 2012; Kim et al. 2017; Mackenzie et al. 2005; Pasinato et al. 2013; Sungur et al. 2015; Quenot et al. 2010; Wang et al. 2017). The three from the grey literature were developed at national levels of respective countries (National Department of Health 2016, 2017; Rice Niamh 2013). Although many studies represented both types of nutritional therapy practice guidelines, the majority (*n* = 17) represented EN, either alone (*n* = 8) or combined with parenteral nutrition (*n* = 9), while nine reported on PN combined with EN and (*n* = 2) (National Department of Health 2017; Rice Niamh 2013) focused on PN only CPGs.

**TABLE 6:** Descriptive characteristics of the included studies.

Author, year and country of origin	Aim or focus of the study and setting	Results on guideline implementation	Conclusions
**Quasi-experimental study**			
Sungur et al. 2015, Pakistan	To determine the effect of the enteral nutrition algorithm on nutritional support in critically ill medical patients in a medical ICU of a university hospital.	40 mechanically ventilated patients divided into two equal groups of 20 (50%) each. Energy intake of study group was 62% of the prescribed energy requirement on the 1st, 68.5% on the 2nd and 63% on the 3rd day, whereas in the historical group 38%, 56.5% and 60% of the prescribed energy requirement were met, respectively. Consumed energy of the historical group on the 1st, 2nd and 3rd day was significantly different (*p* = 0.020).	Use of standard algorithms for EN may be an effective way to meet the nutritional requirements of patients. The study showed that historical group patients required more nutrition than the intervention group.
**Prospective designs**			
Barr et al. 2004, United States of America	Determine if protocol use leads to increased EN, earlier feeding and improved outcomes in medical-surgical ICUs of two teaching hospitals.	200 critically ill adult patients who remained nil per os (NPO) > 48 h after admission to the ICU. 100 patients were enrolled into the pre-implementation group, and 100 patients were enrolled into the post-implementation group. The EN use frequency increased in post-implementation compared to pre-implementation (78% vs. 68%, respectively).	Protocol use increased the patients receiving EN and shortened mechanical ventilation time. About 27% of patients died in the pre-implementation group and 30% died post-implementation.
Cahill et al. 2010a, Canada	Describe current nutrition practices and determine ‘best achievable’ practice relative to evidence-based Critical Care Nutrition CPGs in university hospitals adult medical ICU.	The average use of motility agents and small bowel feeding in mechanically ventilated patients who had high gastric residual volumes was 58.7% (site average range, 0% – 100%) and 14.7% (site average range, 0% – 100%), respectively. There was poor adherence to recommendations for the use of EN formulas enriched with fish oils, glutamine supplementation, timing of supplemental parenteral nutrition and avoidance of soybean oil-based parenteral lipids. Average nutritional adequacy was 59% (site average range, 20.5% – 94.4%) for energy and 60.3% (site average range, 18.6% – 152.5%) for protein.	There is a similar performance gap with respect to pharmaconutrition. Large gaps exist between many recommendations and actual practice with resultant suboptimal nutrition therapy.
Dervan, 2012, United States of America	See possibility to reduce the incidence of overfeeding by implementation of a ‘weaning’ protocol in a10-bed, med-surgical, adult ICU of a tertiary referral teaching hospital.	The study was conducted in patients who were mechanically ventilated for more than 72 h and receiving nutrition support. Overfeeding noted more frequently than underfeeding prior to protocol use (24.6% vs. 19.5% of feeding days) and significantly more often on days when patients were fed by a combination of routes (*p* < 0.05). Post protocol, the incidence of overfeeding reduced almost threefold to 9.1% (*p* < 0.001), and feeding via a combination of routes no longer a significant cause. Underfeeding did not change, and patients being adequately fed increased from 56% to 71% (*p* < 0.001). Post-implementation, overfeeding reduced by threefold, while underfeeding did not change, with the patients being adequately fed.	A ‘weaning’ protocol helps to improve adequate feeding for energy in critically ill patients. Significant causes of underfeeding include GI intolerance, causing interruption for procedures.
Dobson and Scott 2007, United Kingdom	Determine how reliable the updated ICU nurse-led enteral feeding protocol was in medical-surgical ICUs.	Patients who remained PNO > 48 h after their admission to the ICU participated in the study. In all, 90% (*n* = 43) of referrals received by the dietitian met the referral criteria. Absolute compliance with patients receiving correct type and volumes of feed, with a correct feed prescription and an accurate documented weight was just 2% (*n* = 1). Despite this finding, 60% of patients were actually receiving the correct feed regimen. Absolute adherence to the nurse-led EN feeding algorithm was 100%.	Nurse-led feeding algorithm reduced the input of a dietitian on patient feeding algorithm use and empowered nurses to timeouly start NS and safely advance EN towards nutritional goals without the input of a dietician.
Heyland et al. 2004, United States of America	Test the hypothesis that ICUs consistent with the guidelines would have greater success with EN. Intensive care unit affiliated with a registered dietician.	All ICU patients who were in ICU for 72 h and had been mechanically ventilated for 48 h were observed. The observed stay in ICUs ranged from 1.8% to 76.6% (average 43.0%). Intensive care units with a greater than median utilisation of parenteral nutrition (> 17.5% patient days) had a much lower adequacy of enteral nutrition (32.9 vs. 52.7%, *p* < 0.0001). Intensive care units that used a feeding protocol tended to have a higher adequacy of enteral nutrition than those that did not (44.9 vs. 38.5%, *p* = 0.03). Intensive care units that were more consistent with the Canadian CPGs were more likely to successfully feed patients via enteral nutrition.	Consistency with CPGs may translate into better outcomes for critically ill patients receiving nutrition support. Adoption of the Canadian CPGs should lead to improved nutrition support practice in intensive care units.
Heyland, 2015, Canada	Describe experience with implementing protocol and the observed improvements in nutrition intake in an ICU with a multidisciplinary team.	Participants were patients who were mechanically ventilated prior to ICU admission or within the first 48 h, who stayed in the ICU for at least 72 h. Patients at PEP uP sites received 60.1% of their prescribed energy requirements from EN compared to 49.9% of patients from control hospitals (*p* = 0.02). In addition, patients in PEP uP protocol sites received more protein from EN (61.0% vs. 49.7% of prescribed amounts; *p* = 0.01), were more likely to receive protein supplements (71.8% vs. 47.7%; *p* = 0.01) and were more likely to receive > 80% of their protein requirements by day 3 (46.1% vs. 29.3%; *p* = 0.05) compared to patients in control hospitals.	Increased nutrition adequacy could be causally related to improved clinical outcomes of critically ill patients. In the real-life setting, the PEP uP protocol can improve the delivery of EN to critically ill patients.
Pasinato, 2013, Brazil	Evaluate the compliance of septic patients’ nutritional management with enteral nutrition guidelines for critically ill patients. Public, university, and tertiary hospital.	The study was conducted on ICU septic patients, age ≥ 18 years. The patients had a mean age of 63.4 ± 15.1 years, were predominantly male, were diagnosed predominantly with septic shock (56.5%), had a mean intensive care unit stay of 11 (7.2–18.0) days, had 8.2 ± 4.2 SOFA and 24.1 ± 9.6 APACHE II scores and had 39.1% mortality. Enteral nutritional therapy was initiated early in 63% of the patients. Approximately 50% met the caloric and protein goals on the 3rd day of ICU stay, a percentage that decreased to 30% on day 7.	Significant number of septic patients was observed on EEN, but caloric and protein goals at day 3 in ICU were met by only 50%, a percentage that decreased at day 7. It was not possible, however, to show a statistically significant association between meeting the goals and the length of hospital stay, mortality or use of MV.
Quenot et al. 2010, England	Assess adherence to clinical practice guidelines and investigate factors leading to non-adherence. University and/or regional hospitals and general (non-academic) hospitals, mixed medico-surgical and medical ICUs.	Patients receiving mechanical ventilation and without contraindication to initiation of enteral nutrition were included in this study. The median ratio of prescribed or required calories per day was 43 [37–54] at day 1 and increased until day 7. From day 4 until the end of the study, the median ratio was > 80%. The median ratio of delivered/prescribed per day was > 80% for all 7 days from the start of enteral nutrition. A good ratio of calories was actually delivered/prescribed (> 80%) and calories prescribed/required (> 80%), notably after 72 h.	Variables influencing EN and contributing to non-adherence to CPGs: hospital type, local protocol, sedation, vasoactive drugs, number of interruptions and GRV measurement. Satisfactory translation of research and recommendations for EN into practice was observed, but there is also need for a multidisciplinary approach.
Wøien and Bjørk 2006, Norway	Test whether a feeding algorithm could improve the nutritional support of intensive care patients. An ICU staffed for caring for seven patients.	The study participants were patients 20–70 years old who were expected to stay longer than 4 days in ICU. Patients in the intervention group were both prescribed and actually received significantly larger amounts of nutrients than patients in the control group. They also received a larger proportion of their nutrients in the form of EN. In addition, the nutritional support algorithm led to greater consistency in nursing practices with respect to aspiration of gastric content and rate of increment in enteral feeding. Nutrition delivery was higher in intervention group. The algorithm encourages early initiation and rapid increment of NS.	Nurses acted less arbitrarily in executing nutrition orders and aspiration routines for the intervention group. The algorithm resulted in improvements in ICU patients’ nutrition in several areas.
**Retrospective studies**			
Bousie et al. 2016 Europe	Address effects of protocol use on energy and protein adequacy, electrolyte abnormalities, glucose control, staff workload and clinical outcome. Mixed medical-surgical ICU in a tertiary university-affiliated teaching hospital.	In total, 146 mechanically ventilated patients were included (73 patients before and 73 patients after implementation). Before implementation more patients were fed above target (actual caloric intake > 110% of target) than after implementation (during 2–7 days: 12% vs. 3%, *p* = 0.029) without significant reduction of protein intake (daily means during day 2-7: 1.18 g/kg vs. 1.08 g/kg, *p* = 0.09). After implementation only significantly more patients were fed on target on day 6 (47% vs. 67%, *p* = 0.028). Less electrolyte imbalance post-implementation, nurses’ satisfaction improved post protocol and dietitians’ daily workload decreased.	Improved non-significant outcome trends for hospital LOS and for ICU and hospital mortality. Mortality reduction, preventing overfeeding without affecting protein intake and less electrolyte abnormalities were observed after implementation.
Compton et al. 2014, United States of America	Evaluate NS protocol impact on nutrition prescription and delivery in the intensive care unit in a university hospital’s adult med-ICU.	Mechanically ventilated patients, treated in the ICU for a minimum of 5 days were the study participants. After EN protocol implementation EN was started significantly earlier (*p* = 0.007), and EN goals were reached significantly faster (6 vs. 10 days, *p* < 0.001) than before. Prescription of EN on the 1st day of mechanical ventilation increased from 38% before to 54% after (*p* = 0.03) implementation of the protocol. Prescribed and delivered nutrition doses on the first 2 days of mechanical ventilation increased significantly (*p* < 0.001) after the protocol was implemented. Nasojejunal feeding tubes were used in 52% of patients before and 56% of patients after protocol implementation (*p* = 0.63). Jejunal tubes were placed earlier after the protocol was implemented than before (median 5 vs. 6.5 days), and when a jejunal tube was in place, feeding goals were reached faster (median 2 vs. 3 days, *p* = 0.002).	Implementation of an NS protocol significantly improved the EN provision in ICU patients receiving mechanical ventilation. Jejunal feeding tubes were necessary in more than half of the patients, and with a jejunal feeding tube in place, feeding goals were reached rapidly. However, the retrospective approach did not allow assessment of appropriateness of clinical decisions and adherence to the developed protocol.
Kim, 2017, Asia	Evaluate the impact of implementing an EN protocol on the improvement of EN practices and on the clinical outcomes of critically ill patients. Medical and surgical ICU at a university teaching hospital.	A total of 270 ICU adult patients were included, 134 patients before implementation and 136 after implementation of the protocol. Enteral nutritional therapy was initiated earlier (35.8 vs. 87.1 h, *p* = 0.001) and more patients received EN within 24 h (59.6% vs. 41.0%, *p* = 0.002) after implementation of the protocol.	The post-implementation group was given more pro-kinetics and less parenteral nutrition. EN protocol had beneficial effects: EEN, quick achieving target calories, less frequent PN use, and decreasing GI bleeding and diarrhoea.
Kiss, 2012, Switzerland	Determine the impact of using an algorithm on nutrition care outcomes in ICU without a designated dietitian.	Two cohorts of critically ill patients before (*n* = 56) and after (*n* = 56) implementation of an algorithm based on the guidelines published by the Society of Critical Care Medicine and the American Society for Parenteral and Enteral Nutrition guidelines was observed. Significant differences were noticed between the groups for the mean delivery of total energy in the pre- vs. post-implementation *p* < 0.001). There were significant differences between groups for the mean delivery of total energy in the pre- vs. post-implementation group.	This was the first report on algorithm implementation with no dietitian or nutrition support team. Algorithm implementation resulted in improved provision of energy and protein delivery. However, unique dietitian expertise in ICU and specific focus on individualised nutrition support remains ideal and would also increase the adherence to nutrition support guidelines.
Mackenzie, 2005, Canada	Determine whether implementation of an evidence-based nutrition support (NS) protocol could improve EN delivery in a tertiary-care 22-bed medical-surgical referral ICU.	Adult patients who receive either zero EN or PN were included in the study. The percentage of patients who received at least 80% of their estimated energy requirements in ICU increased from 20% before implementation of the NS protocol to 60% after implementation (*p* = 0.001). Post-implementation group received significantly more kcal/kg/d than the pre-implementation group (3.71 kcal/kg/d; 95% confidence interval, 1.64–5.78; *p=* 0.001).	Reduction in the PN use decreased in the post-implementation group. The protocol improved proportion of patients on EN meeting calculated nutrition requirements.
Wang, 2017, Taiwan	Compare pre- and post-implementation outcomes of the feeding protocol, and evaluate the effects of total energy delivery on outcomes in these patients in a tertiary medical centre and general hospital.	The study was conducted on TBI patients, older than 20 years, on EN only, and receiving at least 48 h of mechanical ventilation. Compared with delayed feeding, early feeding was associated with a significant reduction in the rate of mortality (relative risk = 0.35; 95% CI, 0.24–0.50), poor outcome (RR = 0.70; 95% CI, 0.54–0.91) and infectious complications (RR = 0.77; 95% CI, 0.59–0.99). Compared with enteral nutrition, parenteral nutrition showed a slight trend of reduction in the rate of mortality (RR = 0.61; 95% CI, 0.34–1.09), poor outcome (RR = 0.73; 95% CI, 0.51–1.04) and infectious complications (RR = 0.89; 95% CI, 0.66–1.22).	Implementation of the feeding protocol could improve energy intake for critically ill patients; however, it had no beneficial effects on reducing the ICU mortality rate.
**Literature review and expert opinion studies**			
National Department of Health 2016, Republic of South Africa	Provides guidelines and practical strategies for successful implementation of EN regime in adult patients in all public healthcare facilities	Recommendations on nutrition assessment, EN use, handling of complications, monitoring and evaluation of EN for all adult patients in all public healthcare facilities.	Once tolerance is established, there is no need for frequent GRV measurement to avoid inappropriate interruption. A multidisciplinary approach to ensure effective assessment and treatment interventions is needed.
National Department of Health 2017, Republic of South Africa	Provides recommendations based on the best practice of PN management by care workers for all adult patients in all public healthcare facilities.	Recommendations for PN, the roles and responsibilities of the nutrition therapy team in all adult patients receiving parenteral nutrition therapy in government health facilities.	Monitoring ensures adherence to national guidelines. Evaluation allows comprehensive assessment and PN documentation. Parenteral nutritional therapy CPG should ensure evidence-based and standardised PN prescriptions.
Rice 2013, Republic of Ireland	Reviews HPN use and practices, makes proposals for safe, high quality care and equitable access for all suitable candidates in the community health sector.	Deficits identified in coordination, resource planning and clinical governance of the HPN service provision for adult patients who had been mechanically ventilated within 48 h of ICU admission and had been in the ICU for more than 72 h.	GPs and primary care team members lack specialist knowledge of HPN. Recommend for access for all patients in need of HPN.

EN, enteral nutrition; PN, parenteral nutrition; CPGs, clinical practice guidelines; ICU, intensive care unit; LOS, length of stay; NS, nutritional support.

## Thematic findings

The studies have shown that despite the important benefits and positive influence of nutritional therapy guidelines on nutritional practices and clinical course of critical illness, factors exist that hinder their implementation. Therefore, the themes that emerged in this review are discussed under two headings: the efficacy and impact of nutritional therapy practice guidelines and factors influencing implementation (see Table 7).

**TABLE 7:** Themes generated from thematic analysis of results.

Final themes	Meaning	Code list
The efficacy and impact of nutritional therapy practice guidelines	How effective or useful guidelines implementation was (efficacy)	Enteral nutrition (EN) protocols increase EN use, early EN, reaching of feeding goals and shortened mechanical ventilation time. Nurse-led algorithm promoted best practice-based referral criteria so that patients at nutritional risk were referred for tailored dietetic assessment, empowered nurses to start and advance EN timely and safely without dietician input. A significant number of septic patients were started early on EN and there was satisfactory translation of research and recommendations into practice.
	The overall impact of guideline implementation on patient outcomes and health system (impact)	Practice guidelines minimise variations in nutrition practices. Clinical practice guidelines (CPGs) can improve patient clinical outcome, improve quality of life and reduce patient care costs. Consistent nutritional practices improve the provision of nutritional therapy, improve patient care and thus clinical outcomes of critical illness, costs and quality of life.
Factors influencing implementation of nutritional therapy practice guidelines	Positive factors	Factors influencing successful guidelines implementation include awareness of existing guidelines, consensus among clinicians on which guidelines to adopt. A multidisciplinary team involvement, staff education and training, active and passive reminders, presentations by opinion leaders, and discussions during rounds are also recommended for successful guidelines implementation.
	Negative factors	Important deficits include poor coordination, resource planning and clinical governance.

### The efficacy and impact of nutritional therapy guidelines

The studies have provided information on the implementation of nutritional therapy practice guidelines in the form of EN and PN CPGs, protocols and algorithms. Out of five studies that reported on CPGs, three of them were discussing CPGs for EN (National Department of Health 2016; Pasinato et al. 2013; Quenot et al. 2010). According to the authors, EN CPGs can improve patient clinical outcome, improve quality of life and reduce patient care costs (National Department of Health 2016:8) when implemented. A significant number of septic patients were started early on EN, and there was satisfactory translation of research and recommendations into practice (Quenot et al. 2010:1). EN protocols were also found to increase EN use, early EN, reaching of feeding goals and shortened mechanical ventilation time but had no beneficial effects on reducing mortality (Barr et al. 2004:1454; Kim et al. 2017:34; Mackenzie et al. 2005:77). For example, in a study aimed at finding out if EN protocols could improve clinical outcomes, it was found that almost the same number of deaths occurred in both the control and intervention groups, 27% and 30%, respectively (Barr et al. 2004:1454). On the other hand, implementation of algorithms was found to be the standard intervention to operationalise guidelines at the bedside and achieve nutritional requirements (Dobson & Scott 2007:120; Kiss et al. 2012:793; Sungur et al. 2015:813; Wøien & Bjørk 2006:168). Dobson and Scott (2007:120) added that the nurse-led algorithm promoted best practice-based referral criteria so that patients at nutritional risk were referred for tailored dietetic assessment, and also empowered nurses to start and advance EN timeouly and safely without dietician input. Practice guidelines are based on research evidence and contain therapy-specific recommendations intended to minimise variations in nutrition practices (Kiss et al. 2012:793). Consistent nutritional practices improve the provision of nutritional therapy, improve patient care and thus clinical outcomes of critical illness, costs and quality of life (Rice Niamh 2013:12).

### Factors that influence nutrition therapy guidelines implementation

The development and availability of practice guidelines does not necessarily result in a change in clinical practice. Several factors must be considered to successfully implement guidelines (Kiss et al. 2012:793). Factors that influence successful guidelines implementation include awareness of existing guidelines, consensus among clinicians on which guidelines to adopt, and finally adherence by the practitioner to the guidelines (Kiss et al. 2012:793). A multidisciplinary team involvement, staff education and training, active and passive reminders, presentations by opinion leaders and discussions during rounds are also recommended for guidelines implementation (Kiss et al. 2012:793; Rice Niamh 2013). Other important deficits that have been identified in the nutritional service provision for adult patients include coordination, resource planning and clinical governance (Rice Niamh 2013:12).

## Quality assessment results

The results of the MMAT tool show that the published included studies scored between 75% and 100% on the quality score, with five of the studies being 75% and 11 being 100%. The quality scores of the grey literature studies, AACODS, ranged between six (National Department of Health 2016, 2017) and seven (Rice Niamh 2013), which is moderate to high quality. According to the grades of recommendations and levels of evidence, overall, the studies are grade B, IIa and IIb level of evidence as they were non-randomised controlled studies and at least one quasi-experimental study (Mesejo et al. 2011:3). Tables 4, 5a and 5b present quality appraisal results.

## Discussion

This study aimed at examining literature on the extent of nutritional therapy practice guidelines and implementation in the management of critically ill adults. Our study found that adherence to CPGs can improve patient clinical outcome, reduce mechanical ventilation time, ICU and hospital length of stay, save healthcare costs and improve quality of life by improving EN practices and facilitating translation of research and recommendation (Mackenzie et al. 2005:74; National Department of Health 2016:8; Pasinato et al. 2013:22; Quenot et al. 2010:1). This is a crucial finding, particularly in low- and middle-income countries with resource restrictions and a growing burden of critical illness (Gordon, Allorto & Wise 2015:1). Of concern is to notice that 11 of the primary studies were conducted in tertiary institutions and only one compared guideline implementation between academic and community hospitals (which sometimes do not have an ICU). This is sad as critical illness neither starts nor ends within the four walls of ICU. Acutely ill patients are susceptible to malnutrition, and this is common in critically ill patients, occurring in 30% – 50% of hospitalised patients. Malnutrition increases hospital costs and is associated with increased long-term mortality (Wischmeyer 2011). Wischmeyer (2013) reported that a 23-year-old male patient admitted in a small community hospital, complicated on day 3 post-operatively and because he received poor nutrition for a prolonged period, complicated further, was transferred to ICU and died of malnutrition.

In countries such as South Africa, critical care in general faces challenges from changing disease patterns and lack of human and financial resources as these are redirected to primary healthcare and other priorities (Mathivha 2002:1). Unfortunately, the primary care team members and general practitioners lack specialist knowledge, and many hospitals discharging patients on home artificial nutrition do not have a specialist multidisciplinary team (MDTs) (Pasinato et al. 2013; Rice Niamh 2013:29). Another important fact is that most clinicians view nutrition as part of patient care but not as a therapeutic intervention, hence the need for outreach services (Rice Niamh 2013:29).

Although there is growing interest on the implementation of NT practice guidelines in critical care and the practice of providing nutrition to these patients is almost universal, the specifics vary widely from one ICU to another and even among providers (Desai, McClave & Rice 2014:1148). However, it was interesting to find that nurse-led protocols improved NT practice by 100% and relieved the workload of the dieticians and other members of the team (Dobson & Scott 2007:119). This is in agreement with the view of Mauldin and O’Leary-Kelley (2015:24) that nurses are better positioned to screen patients at risk for malnutrition and to work with members of the multidisciplinary team in implementing nutritional therapy plans. Friesecke et al. (2014:204) also believe that adherence to EN guidelines can be improved if ICU nursing staff is responsible for translating it into action with the help of a written algorithm. However, there is still a need for improved knowledge-to-practice translation for all disciplines of healthcare professionals involved with nutritional care of the critically ill patient (Field et al. 2014:2). Coordination, resource planning and clinical governance have been identified as important deficits that need to be considered in the provision of nutritional therapy in adult patients (Rice Niamh 2013:29).

## Strengths

Our study adopted Arksey and O’Malley’s methodological framework, generally recognised as best practice for scoping reviews, and has been adapted to include methodological quality and bias risk assessment. In addition, the included studies scored average or moderate to high in the methodological quality and bias risk assessment, which may help avoid reading of flawed literature and preventing incorporating biased or untrustworthy information into practice. The study also includes non-randomised studies, which are convenient study designs that can suggest possible relationships between the intervention and the outcome. We further searched and included grey literature studies in our review, which is known to reduce publication bias, increase reviews’ comprehensiveness and timeliness and foster a balanced picture of available evidence.

## Study limitations

All published studies adopted non-randomised designs, which are often subject to many types of bias and errors and therefore not considered strong study designs. Studies with inadequate or unclear randomisation tend to overestimate treatment effects up to 40%, which can have a negative impact on the results. Furthermore, non-randomised trials may increase selection bias and having another intervention such as mechanical ventilation, as 11 of included studies have, may cause performance bias. One study reported a withdrawal of more than 50% on day 3 of the study, which means that it had increased attrition bias.

## Recommendations

### Pertaining to research

Based on the results, this study recommends more studies aimed at evaluating existing guideline recommendations for practicability and the development of implementation strategies or models. Further research is recommended for the development of nurse-led bedside EN algorithms that will incorporate the use of pro-kinetics as a strategy to optimise EN delivery in critically ill adult patients; current guidelines recommend EN rather than PN. EN restores gut motility, maintains gastrointestinal integrity and function, minimises translocation of bacteria, improves wound healing and reduces infection risk. Evidence exists that there is a large gap between many recommendations and actual practice, which calls for more research to tailor international and national guidelines. Furthermore, as it may sometimes be unethical to conduct randomised trials when developing nutritional guidelines, protocols that incorporate use of pro-kinetics should be considered as a strategy to optimise EN delivery in critically ill adult patients.

### Pertaining to practice and policy

We recommend the development of bedside algorithms focused on EN and including parenteral nutrition as a supplement to improve the delivery of clinical nutrition in critically ill patients. An algorithm is defined as an operational version of a guideline that is adapted to local requirements and easy to apply in clinical practice. It focuses on specific steps of the nutritional therapy process based on patient-specific conditions and objective assessment.

## Conclusion

Studies have debated that, even in circumstances where implementation was less than perfect, significant improvements were still observed in nutritional practices and nutrition outcomes. We suggest that with greater attention to the implementation of all forms of nutritional therapy practice guidelines customised to suit the local or institutional condition and availability of NST, most critically ill patients will receive the prescribed amounts of nutrients. The consequence will be improvement in nutritional therapy practices, nutritional outcomes, clinical outcomes and reduction in healthcare costs. Satisfactory translation of research and recommendations for EN into practice alone is not enough, there is also a need for a multidisciplinary approach.

### What this paper adds

Evidence on the efficacy and impact of nutritional therapy practice guideline recommendations has increased. Despite this increased evidence, nutritional practices remain varied, leading to suboptimal nutrition delivery. When EN protocols and nurse-led algorithms were implemented, adherence to international and national guidelines was almost 100%, EN was commenced early, nutritional goals were met, mechanical ventilation time decreased. This change in practice allowed the dietitian to dedicate time to more complex cases. Strategies that can enhance adherence to nutritional therapy practice guidelines include structured protocols and algorithms, collaboration, integration, teamwork and fair distribution of resources in the entire trajectory of nutrition care. Whether a patient in need of specialised nutritional therapy is in a tertiary or community health facility should not be an issue. It is the disparities in patient care that should be addressed.
